# Bottom Grinding Increases the Phototrophic Bacteria While Reduces Bacterial Community Stability in Sea Cucumber Cultural Ponds

**DOI:** 10.1111/1758-2229.70357

**Published:** 2026-05-06

**Authors:** Shan Gao, Wei Zhao, Jingwei Jiang, Xiaoyan Guan, Yongjia Pan, Zelong Zhao, Bai Wang, Yao Xiao, Guohan Zhang, Daqian Zhao, Rui Mi, Zunchun Zhou

**Affiliations:** ^1^ Ministry of Agriculture and Rural Affairs Key Lab of Protection and Utilization of Aquatic Germplasm Resource, Liaoning Key Lab of Germplasm Improvement and Fine Seed Breeding of Marine Aquatic Animals, Liaoning Ocean and Fisheries Science Research Institute Dalian Liaoning People's Republic of China; ^2^ Liaoning Normal University Dalian Liaoning People's Republic of China

**Keywords:** community assembly mechanism, co‐occurrence network, cyanobacteria, homogenising dispersal, niche difference

## Abstract

Bottom grinding (BG), which suspends the anaerobic microorganisms deposited at the bottom of the pond through aeration, is a common practice in sea cucumber aquaculture for maintaining water quality. However, little is known about the effects of BG on the environmental microbiome. This study investigated differences in bacterial communities from three niches (surface water, bottom water and sediments) of culture ponds with and without BG operations. Only minor changes in sediment bacterial communities were observed between BG‐treated and control ponds. In contrast, the composition of the bacterial communities in the water was also significantly altered by the BG operation, with an increase in Cyanobacteria and a decrease in Proteobacteria. Additionally, functional prediction revealed increased phototrophy and decreased chemoheterotrophy in aquatic bacterial communities following BG treatment. Co‐occurrence network analysis revealed that bacterial communities in all three niches were more complex but unstable with BG treatment compared to without, indicating some remedial operations for farming practice. Analysis of community assembly mechanisms showed increased stochastic assembly of bacterial communities in all three niches induced by BG treatment. Overall, this study revealed the effects of BG operation on the bacterial communities in culture ponds, providing insights into the ongoing evolution of farming techniques.

## Graphical abstract

Effects of bottom grinding (BG) on bacterial communities in sea cucumber ponds were investigated. BG enhanced Cyanobacteria but reduced Proteobacteria in water bacterial communities. A more complex but unstable bacterial community was observed in ponds after BG. BG improved the migration and stochastic assembly of bacterial communities.

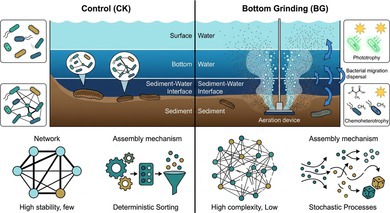

## 1 Introduction

The aquaculture of sea cucumbers ( *Apostichopus japonicus* ) in China has become increasingly important due to its economic, ecological, and nutritional benefits (Yang et al. 2015). Sea cucumbers are rich in nutrients, such as proteins and vitamins, and are renowned for their medicinal properties, including anti‐inflammatory and antioxidant effects (Maskur et al. 2024). Consequently, sea cucumbers are highly valued in domestic and international markets, particularly in Asian countries, where they are considered a delicacy and are used in traditional medicine (Rahman et al. 2020). The high demand has led to a lucrative aquaculture industry in China, which contributes to the local economy and provides livelihoods for many coastal communities (Ru et al. 2019). Additionally, sea cucumber aquaculture can alleviate pressure on wild populations and encourage the sustainable utilisation of marine resources (Baker‐Médard and Ohl 2019). However, the sea cucumber aquaculture sector in China faces challenges such as overfishing, disease outbreaks, and environmental changes (Han et al. 2016). Therefore, sustainable practices and research into farming techniques are essential for the long‐term viability of this industry.

Historically, sea cucumbers have been harvested from the wild using traditional fishing methods prevalent in coastal communities (Li and Zhang 2023). However, the shift towards aquaculture began in the late 20th century, particularly during the 1980s and 1990s, due to overfishing and the decline of wild populations, which prompted the need for sustainable farming practices (Purcell et al. 2012). Advances in breeding and cultivation techniques over the years have improved the yield and quality of farmed sea cucumbers (Zamora et al. 2018). However, as the industry has grown, concerns about sustainability and environmental impacts have also emerged. Issues such as habitat degradation and disease outbreaks have led to calls for more responsible farming practices (Clements et al. 2024). During aquaculture activities, organic matter, uneaten feed, and waste accumulate on the pond bottom over time (Dauda et al. 2019). This accumulation can lead to poor water quality, which is detrimental to sea cucumbers (Hanny et al. 2020). Bottom grinding (BG) involves suspending the anaerobic microorganisms deposited at the bottom of the pond through aeration, thereby improving the hypoxic state at the pond bottom. This is a common practice in aquaculture pond management (Tabrett et al. 2024). Grinding the bottom sediments releases nutrients back into the water column, facilitating their decomposition and reducing the risk of harmful anaerobic conditions (Deng et al. 2022). Implementing effective BG techniques can lead to more sustainable aquaculture practices and improved yields (White et al. 2018).

Bacterial communities play a crucial role in sea cucumber aquaculture, contributing to the health and productivity of the environment (Zhao et al. 2020). These communities are essential for decomposing organic matter and contributing to nutrient cycling within the ecosystem (Lv et al. 2018). Furthermore, these communities can outcompete pathogenic microorganisms for resources, thereby reducing the incidence of disease in sea cucumbers. Therefore, maintaining a healthy and diverse microbial community can enhance the overall health of the aquaculture system (Rajeev et al. 2021). BG treatment can disturb the sediment and mix water in the culture ponds; however, in some aquaculture systems, disturbance of sediments by BG has been shown to affect bacterial communities (Lastauskienė et al. 2021). For instance, one study revealed that BG increased the abundance of sulphate‐reducing and sulphur‐oxidising bacteria in aquaculture ponds (Zhang et al. 2021). The presence of organic‐rich aggregates formed during BG provides a habitat that can support higher bacterial abundance, including potential pathogens (Alfiansah et al. 2018). The effects of BG on bacterial communities in aquaculture ponds are significant and diverse, but have not yet been studied in sea cucumber culture.

This study investigated the effects of BG on bacterial communities in different sea cucumber pond niches (surface water, bottom water and sediment) using high‐throughput sequencing. Two questions were addressed: (i) What effect does BG have on the composition, function and assembly of bacterial communities in sea cucumber ponds? and (ii) How do bacterial communities in different niches respond differently to BG treatment? Understanding these dynamics is essential for optimising aquaculture practices and improving the sustainability of sea cucumber farming.

## 2 Materials and Methods

### 2.1 Studied Aquaculture Ponds and Sample Collection

The sea cucumber ponds that were studied were located in Dalian, China, where aquaculture of *A. japonicus* has continued for over 3 years. These ponds are situated on the coast and cover an area of approximately 8 ha. In these ponds, water exchange depended entirely on natural tides and the sea cucumbers were fed only natural diets; no medication was used during sampling. Three ponds were selected for this study: two were treated with BG and one remained untreated as a control (CK). Samples of surface water (0–10 cm below the water surface, Sur.W), bottom water (0–10 cm above the sediment, Bot.W) and sediment (Sedi.) were collected from each pond over six consecutive days following the BG treatment (once before 6 days for sampling). Approximately 2 L of surface water was sampled each day using a water sampler (Wuhan Shuitiandi Instruments, Wuhan, China), while approximately 1 L of bottom water and 20 g of sediment were sampled simultaneously using a sediment sampler (Beijing Zhongkanance Technology Co. Ltd., Beijing, China). The surface and bottom water samples were immediately filtered through a 0.22 μm pore size polycarbonate membrane filter (142 mm diameter, Millipore, USA) using a peristaltic pump. The filters and sediments were then immediately preserved in liquid nitrogen, transported to our laboratory, and stored at −80°C until DNA extraction.

### 2.2 DNA Extraction and High‐Throughput Sequencing

The E.Z.N.A. DNA Kit (Omega Bio‐Tek, Norcross, GA, USA) was used to extract bacterial DNA from water filters and soil sediments, respectively. The quality of the extracted DNA was assessed using a NanoDrop ND‐1000 Spectrophotometer (NanoDrop, USA) and 1.5% agarose gel electrophoresis. The V3‐V4 region of the bacterial 16S rRNA gene was amplified from the extracted DNA using the 341F (CCTACGGGNGGCWGCAG) and 806R (GGACTACHVGGGTATCTAAT) primers. The PCR and subsequent library construction procedures were consistent with those in our previous studies (Zhou et al. 2021). The constructed sequencing libraries were sequenced on the Illumina NovaSeq 6000 platform using the PE250 strategy at BIOZERN Biotech. Ltd. (Shanghai, China). The resulting sequences were first assigned to the corresponding samples based on the unique barcode attached to the reverse primer. The standard quality control procedure in QIIME2 software was then performed to obtain clean reads, and the amplicon sequence variants (ASVs) were clustered using the DADA2 algorithm (Bokulich et al. 2018). The taxonomy of each ASV was annotated using the SILVA database (release 138) (Quast et al. 2013) to obtain the ASV abundance table. The function of the bacterial communities was then predicted using FAPROTAX software (Louca et al. 2016). Finally, the ASV table was reduced to 43,283 sequences per sample for subsequent analysis.

### 2.3 Statistics Analysis

All statistical analyses were performed using the R v4.2.2 platform (R Core Team 2022) and visualised with the ‘ggplot2’ package (Wickham 2016). Four alpha diversity indices of bacterial communities were calculated using the ‘vegan’ package (Dixon 2003): Chao1, Shannon, Pielou_J, and Pd_Faith. Differences in these indices and in the relative abundance of dominant bacterial phyla, genera, and predicted functional terms between CK and BG ponds were tested using the Wilcoxon rank sum test. Bacterial community composition and function were analysed using the Bray‐Curtis distance, calculated using the ‘vegan’ package. Further analysis was performed using principal coordinate analysis (PCoA) and the Adonis test based on the Bray‐Curtis distance (‘ape’ package) to assess variation in bacterial community structure and function between different niches and treatments (Paradis and Schliep 2019).

The co‐occurrence network of bacterial communities in sea cucumber ponds was constructed based on Spearman's rank correlations between the relative abundances of bacterial ASVs. Only ASVs that were present in at least 60% of samples within the same niche and treatment, with an average relative abundance of at least 0.1%, were included in the correlation analysis (‘psych’ package; Revelle 2022). Co‐occurrences were identified by statistically robust correlations (|correlation coefficient| > 0.8 and Benjamini‐Hochberg adjusted *p*‐value < 0.05) (Steinhauser et al. 2008). Network graphs were visualised, and the topological parameters of the networks were calculated using the igraph package (Csardi and Nepusz 2006). The robustness, vulnerability, and cohesion of the co‐occurrence networks were calculated to evaluate the stability of the bacterial communities, based on a previous study (Yuan et al. 2021). Differences in robustness and cohesion indices between CK and BG ponds were analysed using the Wilcoxon rank sum test.

The neutral community model (NCM) was used to determine the assembly mechanism of bacterial communities in sea cucumber ponds (Sloan et al. 2006). The ASVs in the samples taken from each niche and treatment were divided into three groups depending on whether they occurred above, below or within the 95% confidence interval of the NCM predictions. Additionally, a null model based on the work of Stegen et al. (2013) was performed to elucidate the mechanisms of bacterial community assembly between different niches and treatments. Based on the beta nearest taxon index (betaNTI), the relative contributions of deterministic and stochastic processes to shaping the bacterial community were calculated, and the differences between different habitats and niches were compared. Furthermore, the relative importance of ecological processes in assembling the bacterial community was quantified by combining betaNTI with the Raup‐Crick metric. Deterministic processes can be divided into two categories: homogeneous selection (betaNTI < −2) and heterogeneous selection (betaNTI > 2). Stochastic processes (|betaNTI| ≤ 2) can be assigned to three categories: homogeneous dispersal (RC < −0.95), dispersal limitation (RC > 0.95), and drift (RC ≤ 0.95).

## 3 Results

### 3.1 Diversity of Bacterial Communities Influenced by BG

**FIGURE 1:**
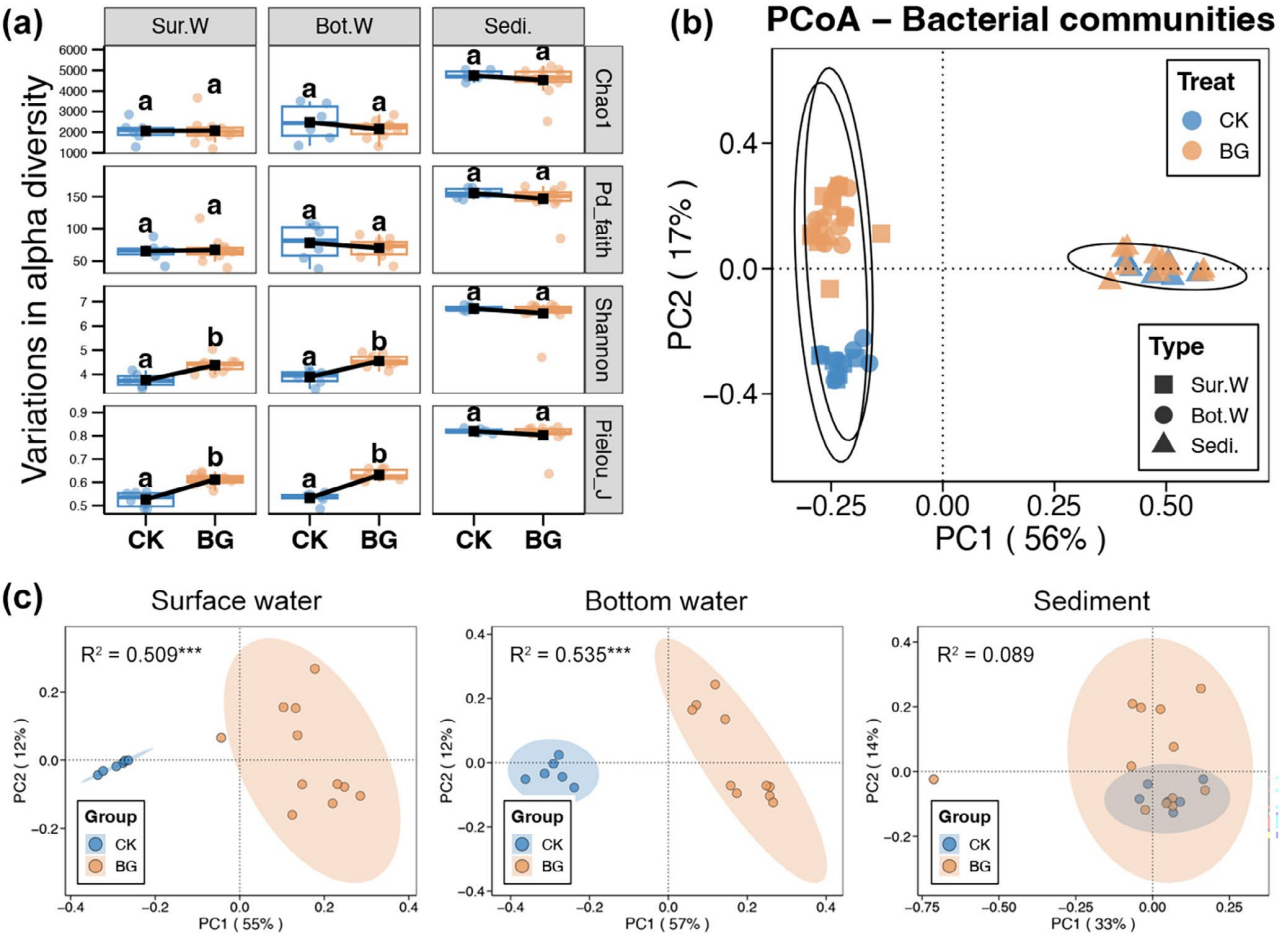
(a) Differences in the alpha diversity indices of bacterial communities in surface water (Sur.W), bottom water (Bot.W), and sediment (Sedi.) between the CK and BG ponds. Different lowercases above the boxes at the same sub‐figure represent significant difference between the CK and BG ponds (Wilcox rank‐sum test, *p* < 0.05). (b) Principal coordinates analysis (PCoA) revealing the variations in the bacterial community structure among different niches and treatments. (c) PCoA and adonis test revealing the variations in the bacterial community structure of surface water, bottom water, and sediment, respectively, between the CK and BG ponds.

A total of 11,230 bacterial ASVs were observed in the sequencing data and were annotated to 61 phyla, 128 classes, 271 orders, 251 families, and 687 genera. Based on a comparison of alpha diversity, it was found that BG did not significantly affect the bacterial communities in the sea cucumber pond sediments (Wilcoxon rank sum test, *p* > 0.05; Figure 1a). Additionally, similar Chao1 and PD Faith indices were observed for the bacterial communities in both the surface and bottom waters of the CK and BG ponds (Wilcoxon rank sum test, *p* > 0.05, Figure 1a). However, it should be noted that BG significantly increased the Shannon and Pielou_J indices in both the surface and bottom waters of the sea cucumber ponds (Wilcoxon rank sum test, *p* < 0.05; Figure 1a). PCoA based on Bray‐Curtis distances showed similar community structures in the sediments of the CK and BG ponds, whereas the community structures in the surface and bottom waters were separated by the PC2 axis (Figure 1b). Furthermore, PCoA plots and the Adonis test for single niches showed that BG significantly affected the community structures of the surface and bottom waters (*p* < 0.05), but not the sediments (*p* > 0.05) (Figure 1c).

### 3.2 Bacterial Community Composition Impact by BG

**FIGURE 2:**
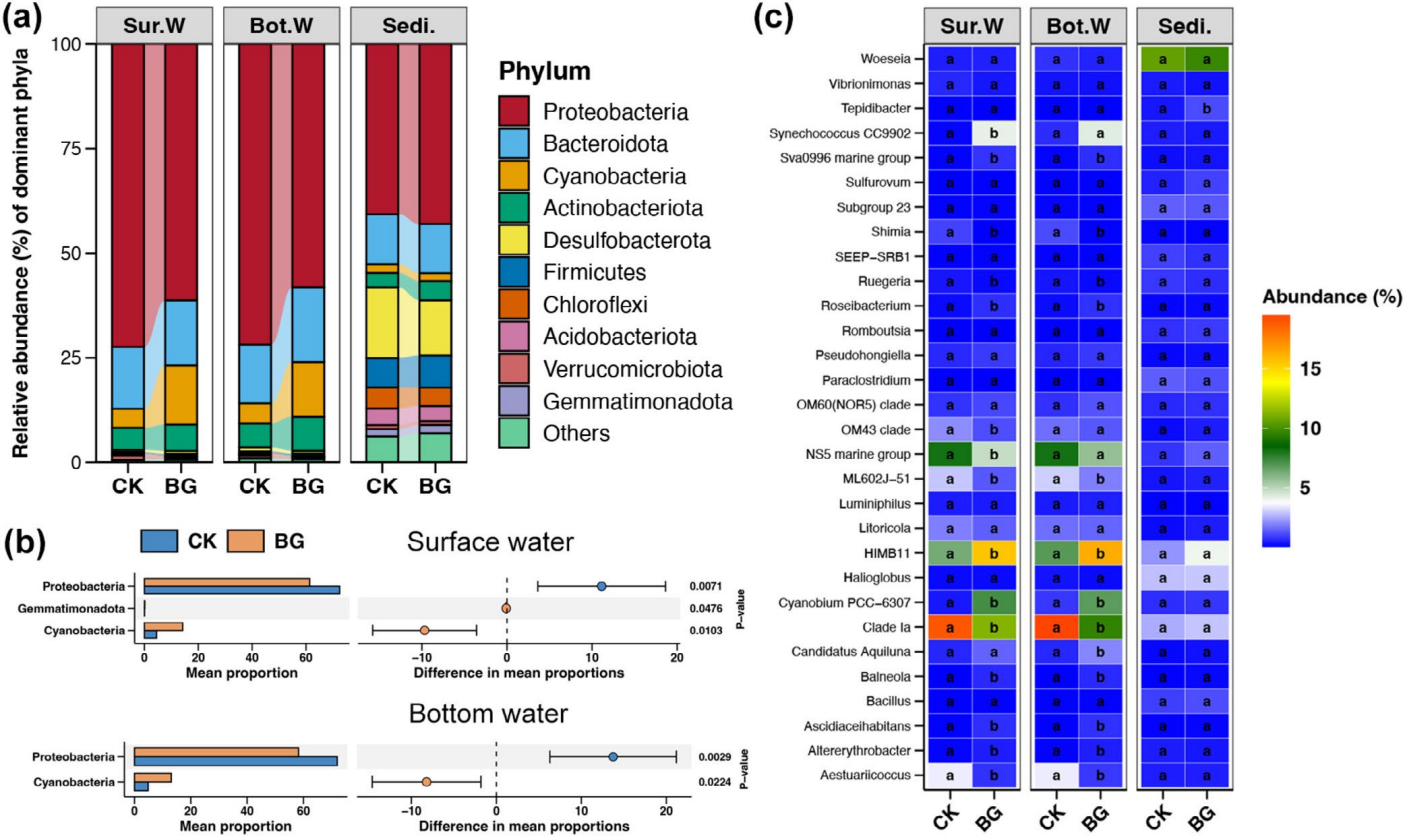
(a) Relative abundance (%) of top 10 abundant bacterial phyla in sea cucumber ponds. (b) Bacterial phyla with significantly different relative abundance in surface and bottom waters, respectively, between CK and BG ponds (Wilcox rank‐sum test, *p* < 0.05). (c) Bubble plot showing the differences in relative abundances of top 30 abundant bacterial genera in surface water, bottom water, and sediments, respectively, between CK and BG ponds. The size and colour of points represent the abundance of corresponding bacterial genus. Different lowercases in bubbles of the same row represent significant difference between the CK and BG ponds (Wilcox rank‐sum test, *p* < 0.05).

Proteobacteria was the dominant bacterial phylum in sea cucumber ponds, whether the sample was from the water or sediment (Figure 2a). Other major bacterial phyla in water samples were Bacteroidota, Cyanobacteria, and Actinobacteriota, while in sediments they were Desulfobacterota, Bacteroidota, and Firmicutes (Figure 2a). Proteobacteria were significantly more abundant in the surface and bottom waters of CK ponds, whereas Cyanobacteria were significantly more abundant in BG waters (Wilcoxon rank sum test, *p* < 0.05; Figure 2b). In contrast, no significant variation in bacterial phyla was observed between CK and BG sediments (Wilcoxon rank sum test, *p* > 0.05; Figure 2b). Similarly, the relative abundances of the major bacterial genera were not significantly different between CK and BG sediments (Wilcoxon rank sum test, *p* > 0.05; Figure 2c). BG significantly increased the relative abundances of *Cyanobium PCC‐6307* and *HIMB11* in sea cucumber pond water, but significantly decreased those of *Clade Ia*, *ML602J‐51*, *Aestuariicoccus*, and *Shimia* (Wilcoxon rank sum test, *p* < 0.05; Figure 2c).

### 3.3 Effects of BG on Bacterial Community Functions

**FIGURE 3:**
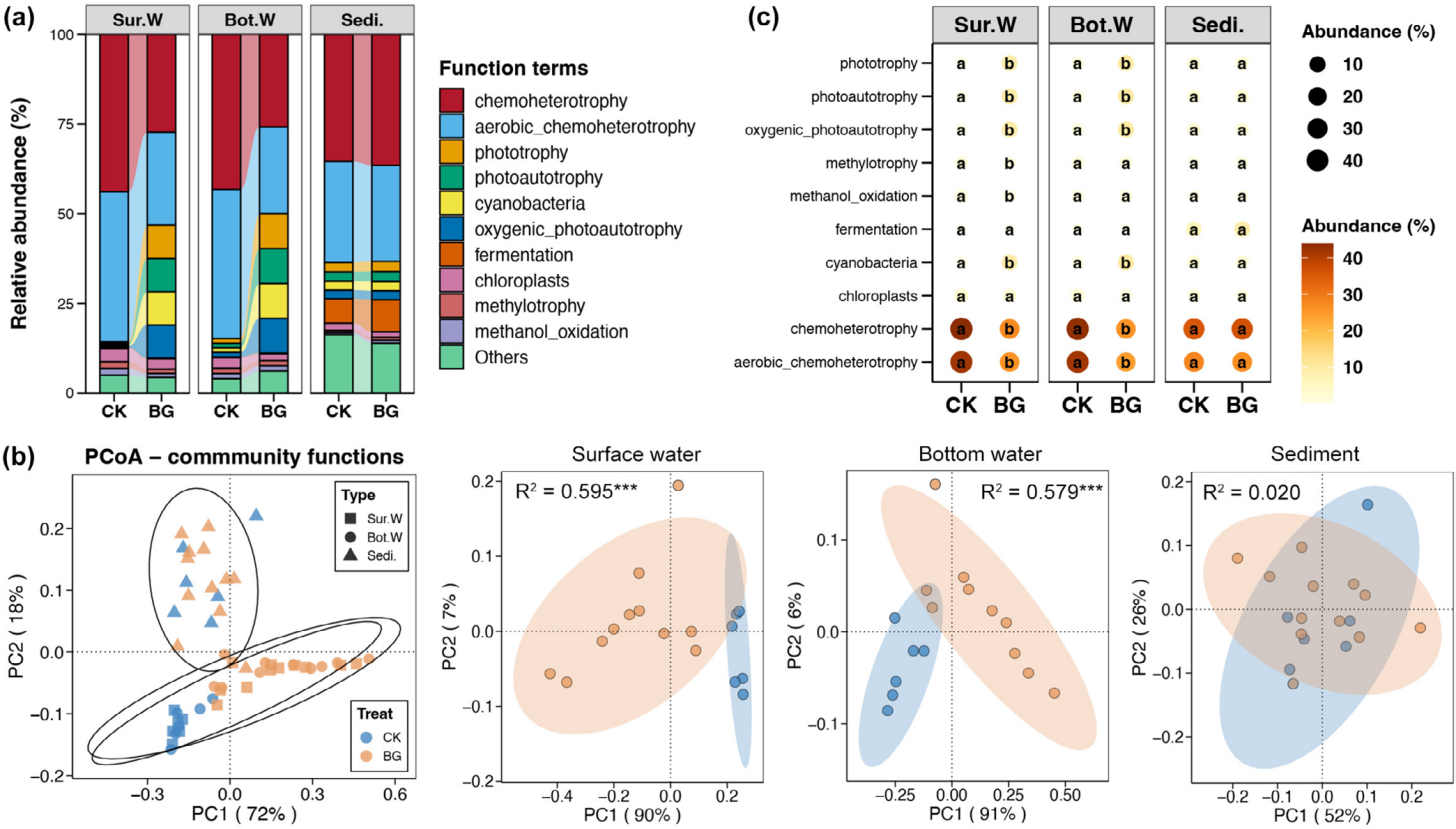
(a) Relative abundance (%) of top 10 abundant functional terms of bacterial communities in sea cucumber ponds. (b) Principal coordinates analysis (PCoA) and adonis test revealing the variations in the bacterial community functions among different niches and treatments. (c) Bubble plot showing the differences in relative abundances of top 10 abundant functional terms of bacterial communities in surface water, bottom water, and sediments, respectively, between CK and BG ponds. The size and colour of points represent the abundance of corresponding functional terms. Different lowercases in bubbles of the same row represent significant difference between the CK and BG ponds (Wilcox rank‐sum test, *p* < 0.05).

According to the FAPROTAX results, chemoheterotrophy was the dominant bacterial community function in sea cucumber ponds (Figure 3a). Similar to the bacterial community structure results, bacterial community function in surface and bottom waters differed significantly between CK and BG ponds (Adonis test, *p* < 0.05), whereas bacterial community function in sediments was not significantly affected by BG treatment (Adonis test, *p* > 0.05) (Figure 3b). No significant difference in bacterial function was also observed between CK and BG ponds in sediments (Wilcoxon rank sum test, *p* < 0.05, Figure 3c). In both surface and bottom waters, BG treatment significantly decreased the relative abundance of chemoheterotrophic bacteria but significantly increased the abundance of phototrophic bacteria (Wilcoxon rank sum test, *p* < 0.05; Figure 3c).

### 3.4 Changes in Co‐Occurrence Network of Bacterial Communities

**FIGURE 4:**
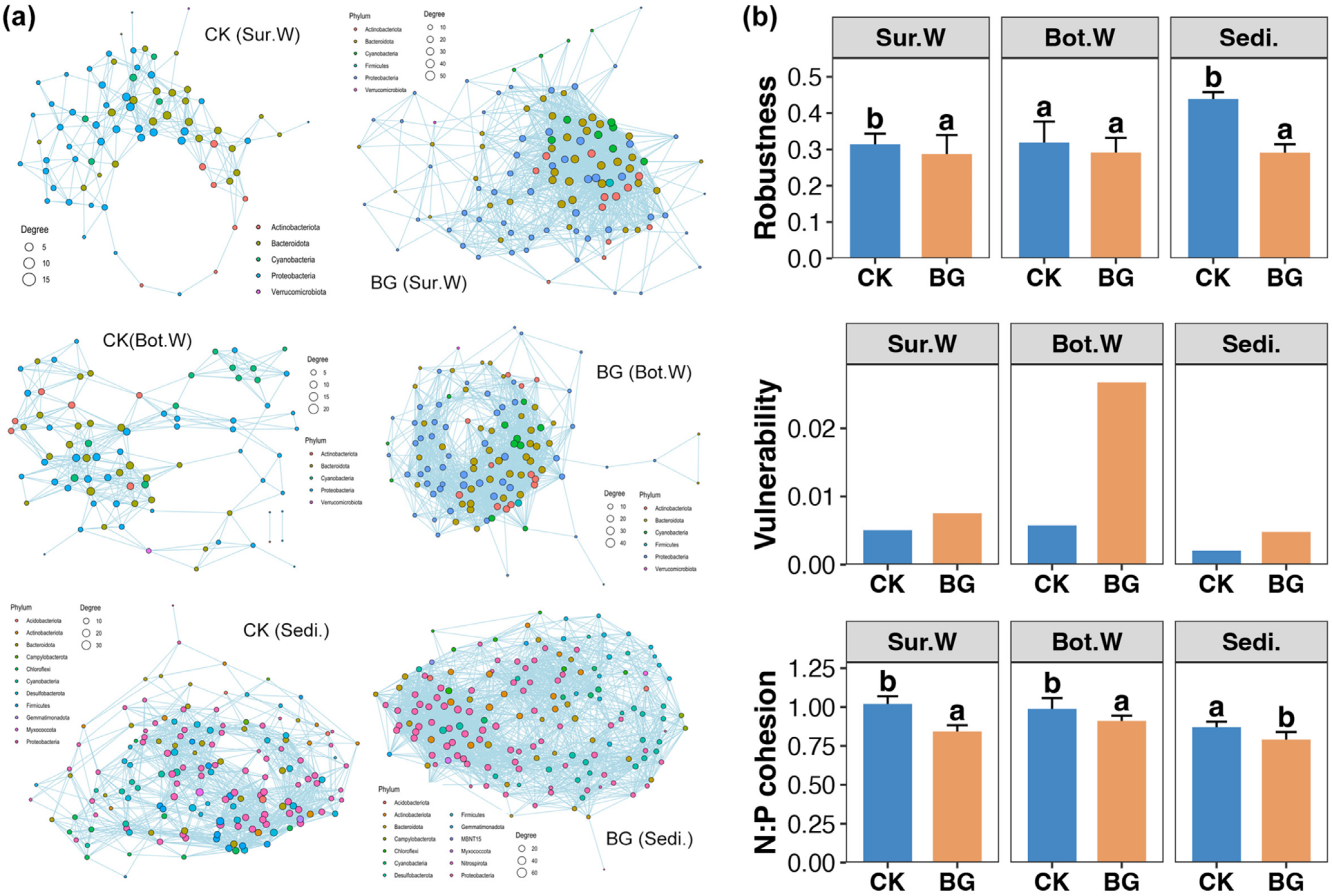
(a) Co‐occurrence networks of bacterial communities in the surface water, bottom water, and sediments of CK and BG ponds. Nodes that belonged to different bacterial phyla are labelled in different colours. (b) Differences in the robustness, vulnerability, and negative: positive cohesion among networks of different niches and treatments. Different lowercase letters above each box in the same sub‐figure represent significant differences between CK and BG ponds (Wilcox rank‐sum test, *p* < 0.05).

Co‐occurrence networks of the bacterial communities in the surface water, bottom water and sediments of the CK and BG ponds were constructed (Figure 4a). All networks followed a power‐law distribution, indicating a non‐random distribution pattern (Table 1). Additionally, the small‐world coefficient was greater than 1 for all networks, suggesting small‐world characteristics (Table 1). As can be seen from the network diagrams, the networks of the BG ponds were clearly more complex than those of the CK ponds in the same niche (Figure 4a). Furthermore, the number of nodes and edges, as well as the average degree, were all higher in the BG ponds than in the CK ponds (Table 1). However, the modularity of networks from the same niche was higher in CK ponds than in BG ponds (Table 1). These results suggest that BG treatment increases the complexity of bacterial community networks in sea cucumber ponds. Conversely, the robustness and cohesion of networks from all three niches were significantly higher in CK ponds than in BG ponds (Wilcoxon rank sum test, *p* < 0.05), while vulnerability was higher in BG ponds than in CK ponds (Figure 4b). These results indicate that BG treatment reduces the stability of bacterial community networks in sea cucumber ponds.

**TABLE 1:** Topological parameters of co‐occurrence networks of bacterial communities in sea cucumber cultural ponds.

Sample type	Treat	Empirical network									Random network			
		Noses	Edges	Modularity	Average degree	Diameter	Density	Average path length	Clustering coefficient	Power‐law model	Modularity (SD)	Average path length (SD)	Clustering coefficient (SD)	Small world coefficient (SD)
Surface water	CK	79	283	0.534	7.165	9	0.092	3.595	0.533	0.988	0.260 (0.387)	2.543 (0.045)	0.112 (0.018)	3.416 (0.372)
	BG	116	1508	0.230	26.000	5	0.226	2.176	0.661	0.808	0.116 (0.161)	1.952 (0.010)	0.320 (0.013)	1.856 (0.047)
Bottom water	CK	83	356	0.549	8.578	9	0.105	3.445	0.607	0.860	0.279 (0.382)	2.394 (0.040)	0.122 (0.018)	3.487 (0.299)
	BG	116	1549	0.290	26.707	6	0.232	2.183	0.616	0.803	0.145 (0.206)	1.927 (0.011)	0.285 (0.012)	1.909 (0.046)
Sediment	CK	164	1330	0.479	16.220	7	0.100	2.945	0.544	0.858	0.239 (0.339)	2.195 (0.025)	0.129 (0.014)	3.148 (0.117)
	BG	168	2712	0.398	32.286	5	0.193	2.120	0.611	0.786	0.207 (0.270)	1.885 (0.008)	0.216 (0.013)	2.520 (0.061)

### 3.5 Assembly Mechanism of Bacterial Communities

**FIGURE 5:**
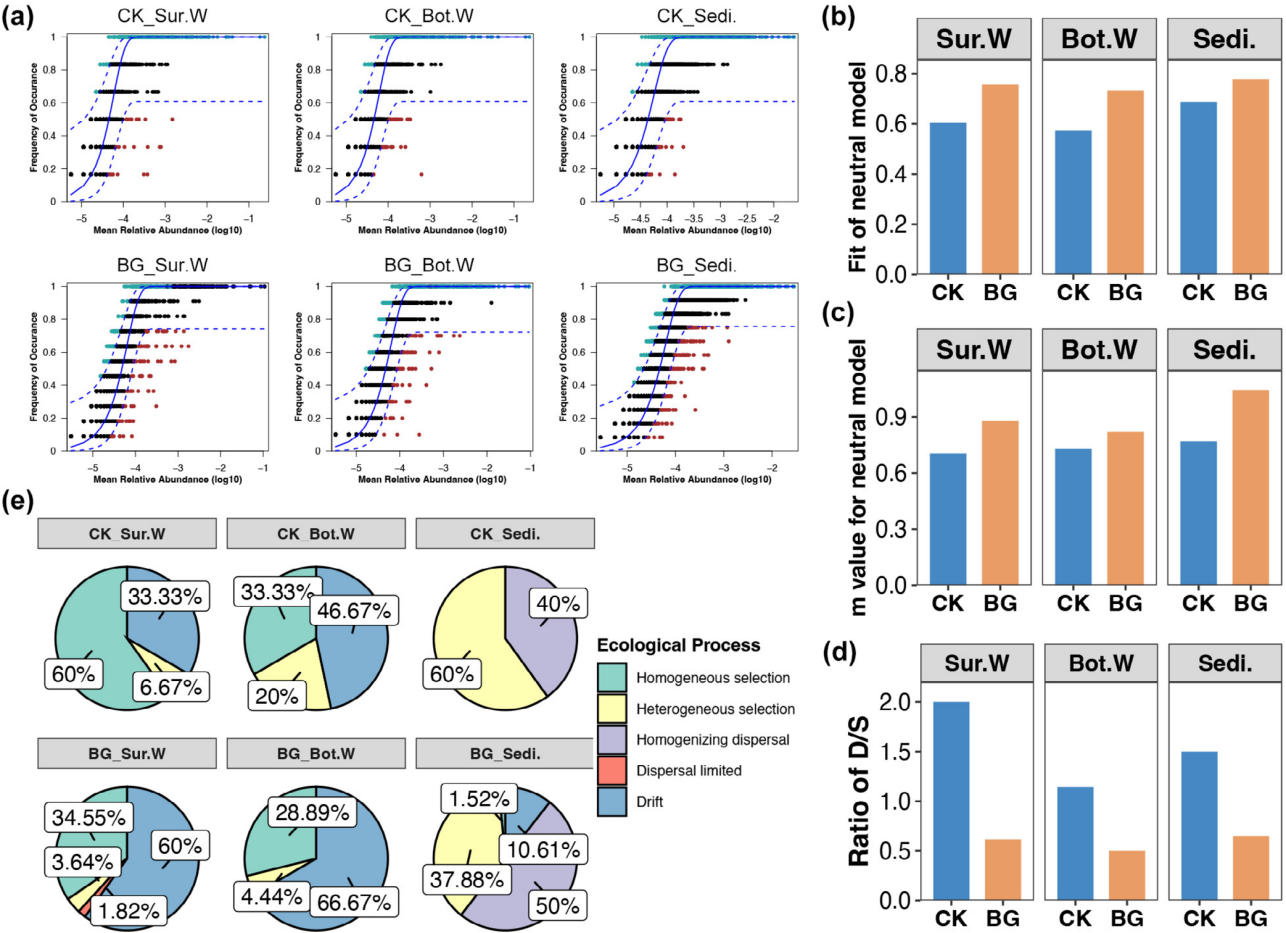
The fitting distribution (a), explained ratio (b), and *m* value (c) of bacterial communities in sea cucumber ponds with different niches and treatments obtained by neutral community models. (d) Ratio of deterministic and stochastic assembly of bacterial communities in sea cucumber ponds with different niches and treatments obtained by the null model. (e) The relative importance of different ecological processes in shaping the bacterial communities of sea cucumber ponds with different niches and treatments obtained by the null model.

NCM was initially conducted to evaluate bacterial community assembly in sea cucumber ponds, identifying ASVs that exhibited neutral distribution patterns (Figure 5a). According to the NCM results, the bacterial communities in the sea cucumber ponds were found to be more influenced by stochastic assembly in the presence of BG (Figure 5b). The m value obtained from NCM also indicated that bacterial ASVs had a higher migratory ability in BG ponds than in CK ponds (Figure 5c). Furthermore, the null model results also showed that stochastic processes played a lesser role in shaping the bacterial communities of BG ponds than CK ponds (Figure 5d). Homogeneous selection and drift were the dominant deterministic and stochastic processes, respectively, for bacterial communities in sea cucumber pond water, while heterogeneous selection and homogenising dispersal played a greater role in sediments (Figure 5e). BG treatment decreased the contribution of selective processes to bacterial community assembly while increasing the role of drift. Additionally, the BG treatment appeared to enhance homogenising dispersal in the bacterial communities of sea cucumber pond sediments (Figure 5e).

## 4 Discussion

Sea cucumber aquaculture ponds harbour diverse bacterial communities that play critical roles in the ecosystem (Zhou et al. 2022). These communities are dominated by certain phyla, such as Proteobacteria, Bacteroidetes and Firmicutes, and their composition varies between different pond compartments (Zhao et al. 2020). This study found similar results, with Proteobacteria being the most dominant phylum (Figure 2a), and different compositions in the water and sediments (Figure 1b). Previous studies have reported that the redox regime and dissolved oxygen levels are the main drivers of bacterial community composition in sea cucumber culture pond sediments (Robinson et al. 2016). The primary purpose of BG treatment is to resuspend sediments and reduce anaerobic conditions. This study was the first to investigate changes in bacterial communities in sea cucumber culture ponds following BG treatment.

Although the direct target of BG treatment is the sediment, our results showed almost no change in the bacterial communities of the sediments in the CK and BG ponds. This was reflected in diversity, composition and function. During BG treatment, soluble substances are released from the sediment into the surrounding water, but no additional substances enter the sediment (Booth et al. 2000). After BG treatment, the conditions of the sediment itself may not change significantly as the large particles settle and the gas rises, remaining in a stable state as before treatment. This may explain why the bacterial community in the sediment did not change significantly after BG treatment.

Bacterial communities in pond water showed significant differences between CK and BG ponds, compared to sediments. Firstly, the evenness of the bacterial communities in the surface and bottom waters increased following BG treatment (Figure 1a). Sea cucumber aquaculture ponds are usually static, with very limited water flow, which restricts the movement of bacterial communities in different areas of the pond (Xu et al. 2019). During the BG period, however, water bodies at different locations in the pond were thoroughly mixed. This interaction and fusion created a more uniform community state. Secondly, *HIMB11* was the bacterial genus that increased the most in relative abundance in both surface and bottom water after BG treatment (Figure 2b). *HIMB11* plays a crucial role in marine biogeochemical cycles, which are essential for maintaining ecological balance and nutrient cycling (Durham et al. 2014). In particular, *HIMB11* is crucial for processing a significant proportion of total carbon in marine environments (Zhang et al. 2021). These characteristics suggest that *HIMB11* may be enriched with nutrients resuspended by BG, and that it may be involved in the transformation of these nutrients in sea cucumber aquaculture ponds. Thirdly, BG treatment significantly increased the number of phototrophic bacteria (especially cyanobacteria) in the water of sea cucumber aquaculture ponds (Figure 3c). Cyanobacteria can fix carbon dioxide to produce organic matter and oxygen (Lucius and Hagemann 2024). This enrichment could primarily be related to the enhanced nutrients in the water due to BG treatment (Richardson et al. 2019). However, it should be noted that over‐enrichment of cyanobacteria can lead to eutrophication, significantly impacting water quality and having negative effects on aquatic life (Lürling et al. 2018).

According to the results of this study, the bacterial communities of all three niches studied exhibited a more complex but unstable co‐occurrence network after BG treatment (Figure 4). Given the stable richness of the bacterial communities, this increase in complexity implies more frequent bacterial interactions (Gao et al. 2022). BG treatment improves the exchange of bacteria in the aquaculture pond, thereby increasing the likelihood of interactions between different bacterial species. The higher bacterial migration in BG ponds revealed by the NCM further supports this view (Figure 5c). However, compared to complexity, network stability may be more critical for sea cucumber farming after BG treatment, as it ensures that associated ecological functions are maintained over time (Zhang et al. 2024). The unstable bacterial communities following BG treatment imply dynamically changing ecological functions and relatively low resilience (Bardgett and Caruso 2020). Therefore, disturbance to the sea cucumber aquaculture pond should be avoided for a period after BG treatment. BG operations should avoid high temperatures or rainy weather and continuously monitor pond water conditions after treatment.

Both the NCM and the null model indicated that operating the BG improved the stochastic assembly of bacterial communities in sea cucumber aquaculture ponds (Figure 5). While both deterministic and stochastic mechanisms influence bacterial communities (Menéndez‐Serra et al. 2023), recent studies have emphasised the predominance of stochastic factors in various aquaculture settings. For instance, one study revealed that the spatial turnover of bacterial communities in shrimp culture ponds was predominantly driven by stochastic processes (Hou, Zhou, et al. 2021). Furthermore, stochasticity has been identified as a critical driver in aquaculture ponds under conditions of high nutrient loading and active water management practices (Niu et al. 2024). These results provide insight into the stochastic assembly of bacterial communities in aquaculture ponds and highlight the importance of understanding microbial dynamics in order to improve aquaculture practices and manage pond ecosystems effectively (Hou, Li, et al. 2021). Furthermore, the increased stochastic assembly of bacterial communities induced by BG treatment was mainly attributed to drift and homogenising dispersal in water and sediments, respectively (Figure 5e). In community assembly theory, dispersal is defined as a stochastic process (Zhou and Ning 2017). In ecology, dispersal is usually natural, but in this study, the dispersal of bacteria in sediments was more likely caused by the deterministic human activity of BG operation. Therefore, it may be necessary to improve current ecological methods when studying the assembly of microbial communities in artificial systems or ecosystems with clear human disturbance. Moreover, the BG operation should not be performed frequently to avoid excessive instability; perhaps conducting it once in the end of summer would be appropriate.

## 5 Conclusions

In conclusion, this study investigated the impact of BG operation on the bacterial communities in sea cucumber ponds. At a fundamental level, it appears that BG treatment does not alter the diversity, composition or function of bacterial communities in the sediment. However, the evenness of water bacterial communities increased significantly following BG treatment. Meanwhile, the composition and function of water bacterial communities varied due to BG operation, as evidenced by an increase in cyanobacteria and phototrophic bacteria, and a decrease in Proteobacteria and chemoheterotrophic bacteria. Additionally, a more complex, yet unstable, bacterial community network was observed in both water and sediments following BG treatment. Furthermore, BG treatment improved the stochastic assembly of bacterial communities in sea cucumber culture ponds by increasing drift and homogenising dispersal in water and sediments, respectively. The future of sea cucumber aquaculture in China looks promising, with ongoing research aimed at improving farming techniques and addressing environmental challenges. The results of this study could help further develop sea cucumber aquaculture while balancing economic growth with environmental sustainability.

## Author Contributions

**Shan Gao:** methodology, formal analysis, writing – original draft. **Wei Zhao:** methodology, formal analysis. **Jingwei Jiang:** conceptualization, supervision, funding acquisition. **Xiaoyan Guan:** investigation. **Yongjia Pan:** investigation. **Zelong Zhao:** writing – review and editing. **Bai Wang:** methodology. **Yao Xiao:** resources. **Guohan Zhang:** investigation. **Daqian Zhao:** investigation. **Rui Mi:** resources. **Zunchun Zhou:** conceptualization, supervision, project administration, writing – review and editing.

## Funding

This work was supported by National Natural Science Foundation of China (U24A200104), Liaoning Revitalization Talents Program (XLYC2203191), Science and Technology Project of Liaoning Province (2023JH1/10200007), Dalian Science and Technology Talent Innovation Support Program (2023RJ007), and Fundamental Research Funds of Liaoning Academy of Agricultural Sciences (2025HQ1304, 2025JCX1008).

## Conflicts of Interest

The authors declare no conflicts of interest.

## Data Availability

The data that support the findings of this study are available on request from the corresponding author. The data are not publicly available due to privacy or ethical restrictions.
